# European Society of Child and Adolescent Psychiatry (ESCAP) practical guidance for clinicians and mental health services regarding child to adult mental health service transitions and managed discharge at the service boundary

**DOI:** 10.1007/s00787-026-03006-6

**Published:** 2026-03-14

**Authors:** Helena Tuomainen, Rebecca Appleton, Gail Asher, Gwendolyn C. Dieleman, Tomislav Franić, Giovanni de Girolamo, Ellie Jackson, Eniko Kiss, Athanasios Maras, Simone Marchini, Fiona McNicholas, Mathilde M. Overbeek, Diane Purper-Ouakil, Anne Marie Råberg Christensen, Paramala Santosh, Ulrike M.E. Schulze, Cathy Street, Leanne Walker, Dieter Wolke, Swaran P. Singh

**Affiliations:** 1 Warwick Medical School, University of Warwick, Coventry, CV47AL UK; 2 NIHR Policy Research Unit in Mental Health, Division of Psychiatry, University College London, London, UK; 3 Derbyshire Healthcare NHS Foundation Trust, Derby, UK; 4 Child and Adolescent Psychiatry/Psychology, Erasmus MC, Rotterdam, The Netherlands; 5 University Hospital of Split, University of Split School of Medicine HR, Split, Croatia; 6 IRCCS Centro San Giovanni di Dio Fatebenefratelli, Brescia, Italy; 7 The Midlands Young Advisors, Associate Development Solutions, Sheffield, UK; 8 Department of Pediatrics, Child and Adolescent Psychiatry Unit, University of Szeged, Szeged, Hungary; 9 ARQ National Psychotrauma Center, Diemen, The Netherlands; 10 Laboratory of Developmental Psychiatry, Faculty of Medicine, Université Libre de Bruxelles, Brussels, Belgium; 11 Child and Adolescent Psychiatry Service, Hôpital Universitaire de Bruxelles, Brussels, Belgium; 12 School of Medicine & Medical Science, University College Dublin, Dublin, Ireland; 13 Lucena CAMHS, SJOG, Dublin, Ireland; 14 Clinical Child and Family Studies, Vrije Universiteit Amsterdam, Amsterdam, The Netherlands; 15 Yulius Mental Health Organization, Dordrecht, The Netherlands; 16 CHU Montpellier, Child and Adolescent Psychiatry (MPEA1), Montpellier, France; 17 INSERM, CESP, Villejuif, France; 18 Child and Adolescent Mental Health Center, Mental health Services, Capital Region of Denmark, Glostrup, Copenhagen, Denmark; 19 Department of Child Psychiatry, King’s College London, London, UK; 20 Centre for Interventional Paediatric Psychopharmacology and Rare Diseases (CIPPRD), Maudsley Hospital, London, UK; 21 HealthTracker Ltd, Gillingham, Kent, UK; 22 Department of Child and Adolescent Psychiatry, Psychosomatics and Psychotherapy, University of Ulm, Ulm, Germany; 23 Department of Psychology, University of Warwick, Coventry, UK

**Keywords:** Clinical guidance, Mental health services, Transitional care, Youth mental health, Service transitions

## Abstract

The European Society for Child and Adolescent Psychiatry (ESCAP) clinical guidance on transition supports care across the child and adolescent mental health service (CAMHS) and adult mental health service (AMHS) boundary throughout Europe. It outlines practices, procedures, and service environments to promote appropriate, safe and timely transition of young people from child and adolescent to adult mental health services or alternative care settings. The guidance addresses planning, decision-making and discharge management at CAMHS and, where needed, continuity in AMHS. The development of the guidance followed established methodological standards for clinical guidance production, combining evidence review, patient and public involvement (PPI), and expert consensus through a structured four-stage process. The guidance is presented in two parts: Part 1 covers six key domains of transition practice, while Part 2 targets service improvement. Intended primarily for clinicians and service managers and providers, the guidance also offers useful information for young people and their families. To enhance local relevance, countries should adapt recommendations to national service and policy contexts.

**Supplementary Information:**

The online version contains supplementary material available at 10.1007/s00787-026-03006-6.

## Introduction

The European Society for Child and Adolescent Psychiatry (ESCAP) clinical guidance on transition aims to support transitional care at the child and adolescent mental health service (CAMHS) and adult mental health service (AMHS) boundary across Europe. It provides guidance regarding practices and procedures, and services and environments designed to promote the appropriate, safe and timely passage of service users across care settings. This includes the planning, decision-making and management regarding the end of care at CAMHS and – if needed – continuity of care in AMHS or another care setting. The guidance is based on the latest evidence associated with mental health service transitions and can support transitional care in any European country.

The guidance covers the basic principles of transition and is thus applicable to transitional care related to most mental health conditions treated in CAMHS. Some mental health conditions will benefit from additional, more bespoke guidance, such as eating disorders [1–3] and attention deficit hyperactivity disorder (ADHD) [4–7].

The guidance is intended for (a) health care practitioners (clinicians) in CAMHS and AMHS and (b) mental health care leaders, managers and service providers. While targeting these professionals, young people using mental health services who may need support from services for adults in the future and parents/caregivers (includes partners and siblings, if appropriate) of young people using mental health services may also benefit from information in this guidance document.

For each country, an adjustment of this guidance to local service and policy context will be necessary. In general, the guidance is intended to facilitate the development of service level protocols and/or checklists. To help with the latter, this guidance has been transformed into a checklist format, available in the Supplement.

## Methodology

The development of this European Society of Child and Adolescent Psychiatry (ESCAP) clinical guidance on CAMHS-AMHS transition followed established methodological principles for clinical guideline development, incorporating both evidence-based approaches and expert consensus [8]. The guidance was developed through a structured four-stage process:

### Stage 1: Framework development

An initial framework and scope for the review and guidance was developed through examination of existing relevant clinical guidelines and guidance documents [9]. This preliminary framework was reviewed and approved by the ESCAP clinical division board to ensure alignment with organisational priorities and clinical needs.

### Stage 2: Evidence synthesis

A comprehensive systematic literature review was conducted to identify, evaluate, and synthesize existing evidence on CAMHS-AMHS transition practices. The methodology and findings of this systematic review have been published separately in the same journal issue [10]. Based on this evidence synthesis, H.T. developed an initial draft of the clinical guidance structured around the temporal trajectory of the transition process: before, during, and after the transition boundary [11–15]. Evidence from studies meeting the review inclusion criteria was synthesized according to which phase of transition it addressed, then evaluated based on its applicability to clinical practice, strength of findings, and potential to inform actionable recommendations for each transition phase. A formal quality appraisal tool was not applied to individual studies.

### Stage 3: Multi-stakeholder consultation

The draft guidance underwent extensive consultation with key stakeholder groups:

#### Clinical consultation

Clinicians from 10 European countries (Belgium, Croatia, Denmark, France, Germany, Hungary, Ireland, Italy, the Netherlands, and the UK) participated in the review process, providing expert feedback on the practical applicability and clinical relevance of the recommendations.

#### Patient and public involvement

Feedback was gathered from two young people and a parent/caregiver with lived experience of mental health service transitions.

#### Professional networks

Additional input was sought from the Clinical Division of ESCAP. The role of the Clinical Division is to promote the quality of professional work and activities in child and adolescent psychiatry and the standing of the profession by supporting the establishment of European practical clinical guidance on relevant topics based on scientific evidence. Members of the Clinical Division cover diverse European countries. They reviewed, and the board of ESCAP approved, the penultimate version of the transition guidance, prior to submission for publication.

### Stage 4: Revision and finalisation

The guidance was revised based on comprehensive feedback collected through structured consultation processes, including written feedback forms distributed to all consultation participants and iterative revision cycles to incorporate recommendations. A transparent process was in place to manage and reconcile any potentially divergent feedback, although no substantive conflicts arose during consultation. A simplified Checklist version of the guidance has also been developed (See Supplement).

## Development team and governance

### Main working group

The clinical guidance development was led by members of the MILESTONE consortium, a collaborative network established through a European Union-funded research programme (2014–2019) focused on transitional care policies and practices across Europe. The consortium comprises clinicians and researchers from both child/adolescent and adult mental health services across eight European countries: Belgium, Croatia, France, Germany, Ireland, Italy, the Netherlands, and the United Kingdom.

The MILESTONE consortium conducted a comprehensive five-year programme of research exploring transitional care through seven interconnected research strands [16], providing the foundational knowledge base for this guidance development.

### Patient and public involvement

Patient and public involvement (PPI) was integral to the guidance development process. Two young people (L.W. and E.J.) and one parent/caregiver (G.A.), all from the United Kingdom with direct experience of mental health service use and transitions, participated as reviewers of the draft guidance document. Their feedback was incorporated through structured review processes, ensuring that the guidance reflects the perspectives and priorities of service users and their families. L.W., a PPI member of the MILESTONE consortium and a Lived Experience Project Support Officer for an NHS Trust, recruited G.A., a Parent Expert by Experience, and E.J., who was part of The Midlands Young Advisors, an autonomous team of young people aged 16–24 from different backgrounds and lived experiences of mental health services.

When adapting this guidance to country-specific service and policy contexts, it is strongly recommended that local PPI members and/or patient advocacy services are actively involved throughout the process.

## Methodological limitations

Several limitations should be considered when interpreting this guidance. Although clinicians from 10 European countries contributed to the consultation, with additional input from the Clinical Division of ESCAP representing a broader European perspective, representation was uneven and some European healthcare systems and service models were not included. The guidance may therefore not fully reflect the diversity of organisational, legal, and resource contexts across Europe.

PPI was limited in both scale and geographical scope (UK only). Despite efforts to recruit contributors from other countries, especially Germany, this was challenging, reflecting the variable development of PPI across Europe. While the input of three members was invaluable, broader representation would have strengthened cultural and systemic applicability.

Given the broad scope of the review, which encompassed diverse study topics, populations, designs, and methodologies, a formal quality appraisal of individual studies was not undertaken; evidence was instead appraised holistically for relevance and practical utility. This pragmatic approach supports clinical applicability but limits the ability to weight recommendations according to graded levels of evidence.

Finally, as the guidance integrates evidence synthesis with expert consensus, it should be viewed as a framework to support best practice rather than a prescriptive or exhaustive standard. Future updates with wider geographical and PPI representation will be important to enhance its robustness and generalisability.

## Overview of guidance

**Fig. 1:**
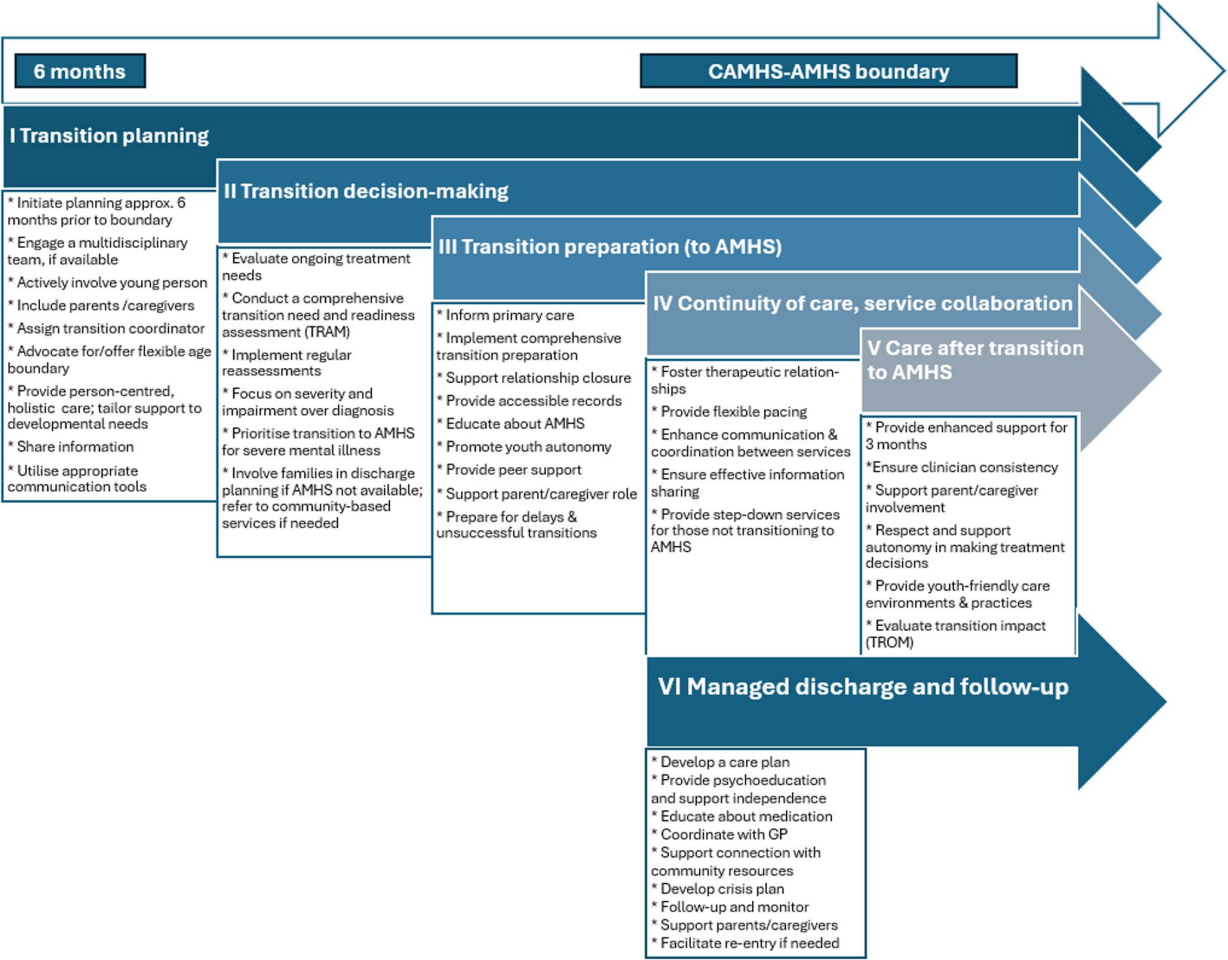
ESCAP transition guidance stages for clinicians, associated with transition planning, preparation and processes

This guidance is in two parts: **Part 1** focuses on the CAMHS (and AMHS) **clinician** and provides guidance linked to (i) transition planning, (ii) decision-making, (iii) preparation, (iv) continuity of care and service collaboration, (v) care after transition to AMHS, and (vi) managed discharge and follow-up. Figure 1 provides an overview of these stages. **Part 2** focuses on **service managers** and how they can support and improve transition processes and care.

## Part 1. Transition planning, preparation and processes

### Transition planning

Transition planning should happen in CAMHS well before the transition boundary and support shared decision making and thorough transition preparation. The service user age at the boundary is 18 years in many countries and services but can range from 16 to 25 years [17].

#### Timing and people involved

1. **Start early**: Allow sufficient time for information sharing, preparation and a gradual transition process. Initiate planning at approximately 6 months before the service boundary for those in care over 12 months. Early planning ensures that young people and their parents/caregivers are better prepared.
2. **Multidisciplinary approach**: If available and feasible, engage a multidisciplinary team of care providers with knowledge of a broad range of services and opportunities in the transition planning process. A multidisciplinary approach helps address young people’s diverse needs at the service boundary and enables smoother transitions and experiences.
3. **Involve young people**: Actively involve young people throughout the transition planning process, seeking their input, preferences and perspectives to promote shared decision-making. They should understand they have choices and control over their care, with clear information about real-world consequences, including written materials they can review later and share with trusted family members and friends. Their decisions should be informed, supported and respected.
  When the young person is too unwell or impaired to participate actively, involve family members or advocates in accordance with relevant legal frameworks and consent requirements. Continue seeking the young person’s views where possible using shorter sessions and alternative communication methods, and revisit decisions when capacity improves.

4. **Include parents/caregivers**: With the young person’s consent, involve parents/caregivers in transition planning. In some circumstances this may not be appropriate (e.g., the young person is not living at home and may not be in contact with their family) and the young person may prefer another trusted person instead. The level of involvement should reflect the young person’s needs and preferences, while recognising that families also need support and guidance to prepare for the shift in responsibilities. Where appropriate, involve parents with lived experience as “navigators” to support others through the process.
  When a young person identifies a trusted person who may pose safeguarding concerns or whose involvement could be detrimental to their care, clinicians should explore the young person’s reasoning sensitively, discuss potential risks, and offer alternative support options, adhering to safeguarding protocols where necessary.

5. **Transition coordination**: Allocate a person in the existing care team (e.g., a nurse) as a point of contact for the young person and their parent/caregiver to facilitate continuity and support the coordination of care during the transition process.
  Where available, involve navigation services to reduce barriers, streamline care pathways, connect families to appropriate supports and advocate for them within the mental health system (i.e., dedicated personnel who support young people and families in understanding service options, coordinating referrals and navigating complex healthcare and social systems).

6. **Increase service flexibility in timing**: Advocate for or, if feasible within regulations, offer flexibility in transition age boundaries, allowing CAMHS care to extend, if needed, or a period of parallel care in CAMHS/AMHS.

#### Care and support

7. **Foster respectful, person-centred care**: Treat young people and their parents/caregivers with respect, valuing their preferences and perspectives throughout the transition process. This includes supporting informed choices regarding destination services, particularly when moving away for college or university.

8. **Developmentally appropriate support**: Tailor transition planning to the young person’s maturity, cognitive abilities, social context and personal circumstances, not just their age. Young people with co-morbid chronic physical health conditions may be navigating multiple transitions - careful consideration of sequencing of these is needed.
  Young people with developmental disorders (e.g., intellectual disability, Autism Spectrum Disorder) may benefit from extended timelines, well-communicated and clearly explained advance notice of changes, structured preparation such as pre-visits, sensory considerations, involvement of specialist services, and continuity of key support persons where possible.

9. **Holistic perspective**: Take a whole person approach by incorporating the young person’s goals, aspirations (e.g., plans to move away from home) and broader life context – such as education, employment, health, housing, relationships and legal circumstances – into transition planning. Planning should be recovery-oriented and focused on supporting functional and social outcomes, recognising emerging adults as a distinct group with unique and evolving needs.

#### Information and communication

10. **Consider best communication tools**: Leverage digital communication tools (e.g., texts, emails, video consultations) to improve accessibility, trust and continuity during transitions.
11. **Information sharing:**
  **Content and scope** - Provide young people with a clearly explained written synthesis of their diagnosis, a comprehensive overview that includes key developmental, social and clinical factors, their strengths, prognostic considerations, and treatment history. Include key information about the transition process, including potential new services, clinical teams and locations. Information resources should be age-appropriate, use accessible language, and provide definitions for any medical terms. They should explain differences between child and adult services, expectations for increased autonomy, follow-up plans and available community and emergency supports.
  Acknowledge and address any uncertainties around diagnosis, as these can affect confidence and access to care. Offer clear explanations and reassurance about current diagnoses, medications, evolving treatment pathways and the possibility that diagnoses may change over time. Ensure information is available in a variety of formats and languages as required.
  **Timing and alternatives** - Information should be shared in a timely way, allowing space for reflection and discussion. Where transition to AMHS is not appropriate, provide information about alternative supports, including voluntary and community-based services (see also *Community service/resource linkage under VI Managed discharge)*.

### Transition decision-making

This section focuses specifically on what factors need to be considered when making the decision about transition.

#### Evaluation of treatment needs

12. Evaluate ongoing treatment needs: Assess symptom severity, functional impairment, disorder progression, and comorbidities, incorporating insights from the young person’s comprehensive assessment including developmental trajectory, personal and environmental resources, and prognostic indicators, to determine the most appropriate care setting (e.g., AMHS, CAMHS, general practitioner [GP] or voluntary sector). Promote independence well before the transition boundary and involve the young person as an equal partner in decision-making, offering clear options for ongoing support.
13. Comprehensive transition need and readiness evaluation: Assess the young person’s readiness and need for transition using a validated standardised tool such as the Transition Readiness and Appropriateness Measure (TRAM) [18, 19] (available via s.p.singh@warwick.ac.uk). This multidimensional tool incorporates the perspectives of the young person, parent/caregiver and clinician to identify key concerns, evaluate the severity of psychopathology, and determine the appropriateness of transition to ongoing care in AMHS.
  Recognising and discussing differing perspectives supports shared decision-making and contributes to better transition outcomes – especially for those with an identified need for continued treatment. The following sections are part of the TRAM:
  - **Diagnosis and symptom severity**: The nature and severity of the young person’s diagnosis and symptom severity help determine whether ongoing specialist input (e.g., AMHS) is needed or if support from community or voluntary services is more appropriate.
  - **Risk factors**: Individual’s risk for harm to self or others, substance use or other risk factors that may require specialised services in the adult system.
  - **Functional ability**: Individual’s daily functioning, self-care abilities and independence skills necessary for navigating adult services.
  - **Psychosocial situation**: Individual’s support system, living situation, educational/vocational status and other psychosocial factors that may impact their transition to adult services.
  - **Engagement and insight**: Individual’s level of engagement with treatment, insight into their condition and willingness to continue treatment in the adult system.
14. **Leverage assessment findings**: Integrate findings from self-reports and assessments into care plans. Address discrepancies and provide additional support as needed.
15. **Regular reassessments**: As mental health problems can fluctuate over time, implement regular reassessments to monitor changes in symptom severity and treatment needs. This can help identify individuals who may no longer require specialist services and could be appropriately discharged or transitioned to lower levels of care.

#### Decision-making

16. **Consider severity or impairment over diagnosis**: Assessments should focus on the overall level of impairment, risk and treatment needs, rather than solely on the diagnosis. Bear in mind the dimensional aspect of disorders.
17.  **Prioritise transition to AMHS for severe mental illness**: Young people with severe conditions like psychosis, bipolar disorder, severe symptoms of depression, anxiety or post-traumatic stress disorder (PTSD), personality disorder, eating disorder, a psychiatric disorder with comorbidity (e.g., type-1 diabetes), suicidal thoughts, self-harm or requiring specialist medication management should be prioritised for transition to AMHS.
18.  **Involve families in discharge planning** *(see also Section VI for full details on Managed Discharge)*: This is especially important if adult services are unavailable (e.g., due to long waiting times) and young people and their parents/caregivers report a need for ongoing treatment. Clinicians should strongly encourage the involvement of young people and parents/caregivers in discharge planning, valuing their input and ensuring shared decision-making. If indicated, refer the young person to or actively support them in connecting with community-based services, e.g., voluntary sector advice and counselling.

### Transition preparation

This section focuses primarily on transition to AMHS while acknowledging that not all young people continue in adult services.

19. ** Inform primary care**: If agreed with the young person, inform their primary-care physician (GP and/or paediatrician) of the transition decision early on.
20. ** Comprehensive preparation**: Develop and implement comprehensive transition preparation to help young people feel ready and address any feelings of uncertainty.
  Together with the young person, develop **individualised care and transition plans** focusing on the young person’s goals of functioning, incorporating assessment findings and action plans.
  Provide the name of the person they are likely to see at the first appointment at AMHS and, where possible, facilitate a face-to-face handover with a direct introduction to the new clinician. Check out with the young person any concerns they may have about new therapeutic relationships (e.g., less frequent appointments) and explore with them possible coping strategies for the next stage.
  This should include preparation for shifting from family-centred care in CAMHS to person-centred care in AMHS; coping strategies for potential waiting periods or longer journey times/travel requirements; discharge to primary care, depending on transition decision outcomes.
  Include structured preparatory work that equips young people with age-appropriate self-management skills, such as psychoeducation, harm-reduction strategies, and coping mechanisms. This can help reduce the risk of maladaptive coping strategies that can emerge under stress.
21.  **CAMHS relationship closure**: Plan the ending with the CAMHS clinician well in advance and conduct it in a (gradual) manner appropriate to the young person’s individual needs and circumstances. Review progress, acknowledge achievements and create space and time for the young person to process emotions around the transition to avoid feelings of abandonment.
  Recognise that not all young people will want a gradual closure - some may prefer a clear ending or choose to take a break from therapeutic input during the transition.
22.  **Provide accessible records**: Equip the young person with easily accessible (electronic if system is sufficiently secure) documentation that can be brought along to the first appointment describing their diagnosis formulation with risks and strengths, care history, prescribed medications, needs and preferences.
23.  **Promote youth engagement and autonomy**: Encourage young people to take an active role in their care while still in CAMHS, including, where appropriate, managing medication. This helps foster independence and autonomy in AMHS, or self-management if discharged, with parent/caregiver support available as needed.
24.  **Peer support**: Facilitate opportunities for young people to connect with peers who have experienced the transition process. Where available and appropriate, signpost to recognised peer support groups, including those offered online or by voluntary/community organisations. Peer mentors help demystify adult services, model coping strategies, and provide valuable encouragement during preparation for transition.
25.  **Support parent’s/caregiver’s roles**: Recognise the significant support roles caregivers often take on during and after the transition. Provide guidance and resources to help them navigate their changing roles in supporting their child’s independence and self-management. Inform parents/caregivers about local carers’ services and support groups.
  Prepare them for the shift from family-centred to person centred care. Support options for parents to accompany the young person to their first appointment in AMHS if appropriate, while also developing alternate plans for situations where the young person prefers to engage independently or declines parental involvement after reaching the age of majority.
26.  **Contingency plan**: Prepare for possible delays, unsuccessful transitions, problems with medication (e.g., side effects) or mental health crises by outlining steps the young person can take to manage their mental health and seek support during the interim, including providing emergency contact numbers and details of crisis services. Be clear that transitions may not always go as planned - delays or disruptions are not uncommon; acknowledging this helps set realistic expectations, reduces distress, and helps young people cope.

### Continuity of care and service collaboration

For ensuring continuity of care at the transition boundary, the following actions are recommended:

27. ** Therapeutic relationships**: Recognise the value of existing relationships and support continuity by:
  1. Involving CAMHS clinicians in introductory meetings with AMHS clinicians;
  2. Joint meetings between CAMHS and AMHS teams;
  3. Engaging a key worker who works across both services.
  Joint meetings and shared key workers require buy-in from both services, which managers should facilitate *(see Part 2)*.
28. ** Flexible pacing**: Tailor the transition pace to individual needs, acknowledging that the time required to build trust can vary significantly between young people.
29.  **Enhance communication and coordination**: Foster collaboration among CAMHS, AMHS, primary care and/or other relevant services (e.g., education, social care, vocational and community-based supports) to ensure seamless transition and consistent care. This includes:
  1. Clear, detailed handovers that clearly outline the young person’s needs;
  2. Verification that AMHS or the receiving service is prepared and able to meet those needs;
  3. Timely AMHS assessment of the young person’s needs, service eligibility, and appropriate support options, with clear communication about whether and when care will be provided;
  4. Prompt referral to another appropriate service when necessary to avoid disengagement or deterioration during waiting periods.
30.  **Ensure effective information sharing**: Ensure timely transfer of up-to-date information, e.g., medical records, prescribed medication and treatment history, to the receiving service or provider. Share this documentation with the young person and their parent/caregiver for transparency and shared understanding. Consider using a patient-held communication booklet and other direct methods (e.g., letters, digital tools) to support information flow between services, including with primary care/GP.
31. ** Provide step-down services**: Not all young people will require ongoing mental health care after leaving CAMHS and a significant proportion will not transition to AMHS. For those who show improvement in their mental health but may still need some level of care, consider organising less intensive support during the initial transition period. This could include less frequent check-ins, referrals to community-based resources or time-limited follow-up appointments to monitor stability.
  Effective step-down requires knowledge of locally available options and verification that receiving services have the capacity and competence to meet the young person’s needs.

### Care after transition to AMHS

In the early stages after a young person has transitioned to AMHS, the following steps are recommended:

32. ** Enhanced support**: Ensure the young people has attended at least one AMHS appointment before CAMHS discharge. AMHS should follow up with young people who do not engage with scheduled appointments within the first three months post-transition.
33. ** Clinician consistency**: Ensure the same clinician sees the young person for the first two appointments to build rapport and reduce anxiety. For young people requiring weekly support, extend this consistency to 3–4 initial appointments.
34. ** Choice about parent/caregiver involvement**: Allow and support parent/caregiver involvement if the young person wishes for them to be involved.
35. ** Autonomy in treatment decisions**: Respect the young person’s right to make their own treatment decisions. Be mindful that many may not be used to having full autonomy and could need additional support and guidance to navigate choices confidently. Encouraging shared decision-making can help build independence and strengthen their role in managing their own care.
36. ** Provide youth-friendly care environments and practices**: Establish physical care environments geared toward young adults, and flexibility in modes of connection and communication, e.g., video consultations and text messaging.
37. ** Conduct a comprehensive evaluation of the impact of transition**: Assess the impact of transition on mental health outcomes from multiple perspectives using a validated standardised tool such as the Transition Outcome Measure (TROM) which matches the TRAM [18, 19] (available via s.p.singh@warwick.ac.uk). Conduct this within 4–6 months of transition.

### Managed discharge and follow-up

If a young person is not eligible for transition to AMHS or to continue in CAMHS, and is discharged back to primary care, the core elements of a managed discharge process should include the following:

38. ** Comprehensive assessment and care planning**: Conduct a thorough assessment to identify the young person’s ongoing mental health needs, strengths, functional impairments and required community supports. Develop a care plan that outlines specific goals, interventions and resources to address these needs.
39. ** Psychoeducation and self-management**: Provide clear, age-appropriate psychoeducation to the young person and their parents/caregivers about their mental health condition, coping and self-management strategies, and when and where to seek help if symptoms re-emerge or worsen.
  Support independence by equipping the young person with tools and resources – including organisational aids such as apps and symptom trackers – to help manage symptoms and medications effectively.
40. ** Medication management**: If the young person is still taking medication, establish who will take responsibility for this (e.g., GP) and provide comprehensive information including indication, expected effects and side effects, treatment duration, alternatives if not tolerated or effective, and, if appropriate, deprescribing guidance.
  Provide this information to both the GP and the young person, and where appropriate, their parents/carers. Ensure all parties know what to do if they experience any adverse effects.
41. ** Primary care coordination**: Establish close coordination and communication with the young person’s primary care provider (e.g., GP). Where locally available, consider shared care models involving primary care and/or community-based organisations to provide comprehensive support. This may include remote consultation arrangements using digital platforms that enable communication between GPs and specialist services.
  Ensure the transfer of relevant medical records, treatment history and the care plan to facilitate continuity of care.
42. ** Community service/resource linkage**: Identify and actively support the young person to connect with appropriate community-based resources, such as peer support groups, counselling services, vocational training, or social services. Help facilitate initial contact through joint calls, accompanied visits, or warm introductions rather than simply providing leaflets or contact details. Develop strong relationships with community-based services through regular meetings, training and support to enable effective facilitated connections.
43. ** Crisis prevention and management**: Develop a crisis prevention and management plan that outlines steps for the young person and their parents/caregivers to take in case of a mental health crisis or relapse. This should include clear pathways for re-accessing mental health services if needed.
44. ** Follow-up and monitoring**: Young people may experience a clinically significant increase in mental health problems after leaving CAMHS. Therefore, ensure a system is in place for structured follow-up and monitoring, either through primary care or community-based services, to track the young person’s progress, identify any emerging concerns and provide ongoing support and adjustments to the care plan as needed.
45. ** Parent/caregiver support**: Recognise the significant support roles that caregivers often take on during transition and after discharge. Provide ongoing support and education to the young person’s parents/caregivers, to help caregivers navigate their changing roles and support their child’s independence and self-management. Inform/connect parents/caregivers to local carers’ services and support groups.
46. ** Pathway for rapid re-entry**: Maintain open communication with the young person and parents/caregivers to facilitate rapid re-entry into appropriate mental health services if needed, minimising delays or disruptions in care.

## Part 2. Service level guidance

### Service improvement and advocacy

At the mental health service level (CAMHS and AMHS), service managers and senior leadership are recommended to implement the following:

47. ** Allocate sufficient budget and resources**: Ensure appropriate funding and resources for transition activities, e.g., for transition coordinators (from existing care team), joint working time or clinics, and evaluation tools.
48. ** Involve young people in service design**: Actively engage young people (and their parents/caregivers) in the co-design and delivery of transition-related care. Their input helps ensure services are youth-friendly, responsive to their needs and developmentally appropriate.
  A welcoming, age-appropriate environment can support engagement and comfort. Young people often appreciate features such as free WIFI, a comfortable waiting area, a cosy communal space and access to a quiet room for privacy and downtime.
49. ** Establish shared responsibility and ownership for transition**: Promote shared responsibility between CAMHS and AMHS for transition outcomes through genuine inter-service collaboration.
  Engage AMHS early via outreach and introductory meetings, with focus on supporting continued care rather than assessing suitability. Implement collaborative strategies such as joint clinics, shared transition meetings, or parallel care periods. Foster regular inter-service communication with clearly defined responsibilities and mutual accountability for successful transitions.
50. ** Implement transition protocols**: Implement transition guidelines addressing the specific challenges and needs within your local or regional context. Ensure protocols cover key aspects such as early transition planning, age boundaries, information sharing, continuity of care and involvement of young people and parents/caregivers. Regularly review and update protocols based on emerging evidence and best practices.
51. ** Pre-transition monitoring and planning**: Establish a systematic process to identify young people approaching the service age limit at least 6–12 months in advance. Ensure they receive a timely structured transition-readiness assessment approximately 6 months before reaching the service’s upper age boundary.
52. ** Improve service accessibility and navigation**: Locate clinics in areas that are easily accessible by public transport to accommodate young people who may not drive. Ensure service information is current and easy to find, e.g., through regularly updated websites or directories. Strengthen inter-service networks to simplify referrals.
53. ** Provide appropriate training and resources**: Ensure clinicians working with young people in CAMHS *and AMHS* are equipped with appropriate training and resources for delivering transitional care. This should include knowledge of legal frameworks, patient rights, age-specific developmental needs and the skills required to deliver youth-focused, collaborative, and flexible transitional care. Training should ideally be delivered *jointly across CAMHS and AMHS* to ensure consistency and promote collaborative working during the transition period.
54. ** Support continuous improvement through feedback and follow-up**: Regularly monitor and refine transition processes, by incorporating structured follow-ups – ideally at 6 months post-transition – as standard practice. Equip clinicians with validated tools such as the On Your Own Feet – Transition Experiences Scale (OYOF-TES) and the End of Care version (OYOF-EOC) [20] to systematically capture the experiences of young people and their parents/caregivers and identify gaps in service.
55. ** Collaborate with primary care**: Strengthen collaboration and communication with primary care providers, equipping them with resources and training to support young people’s mental health needs. Explore shared care models, designated GP liaison roles, or nurse-led interventions in primary care settings.
56.  **Advocate for system improvements**: Address barriers like rigid timelines, resource constraints, and long waiting lists through policy reform and funding advocacy.

## Supplementary Information

Below is the link to the electronic supplementary material.

Supplementary Material 1

## Data Availability

No datasets were generated or analysed during the current study.
