# Supplementary material for: European Society of Child and Adolescent Psychiatry (ESCAP) practical guidance for clinicians and mental health services regarding child to adult mental health service transitions and managed discharge at the service boundary
Source: Eur Child Adolesc Psychiatry. 2026 Mar 14;35(4):1357–68. doi: 10.1007/s00787-026-03006-6 (PMC13219194; doi:10.1007/s00787-026-03006-6)
Supplement: Supplementary file 1 — Supplementary Material 1 [file 787_2026_3006_MOESM1_ESM.docx]

# European Society of Child and Adolescent Psychiatry (ESCAP) practical guidance for clinicians and mental health services regarding child to adult mental health service transitions and managed discharge at the service boundary

# Checklist

# Part 1. Transition planning, preparation and processes

# I Transition planning

## 1. Initiate early planning

- Begin transition planning approximately six months before the service boundary for individuals in care for over 12 months.

## 2. Engage a multidisciplinary team

- Involve a multidisciplinary team with expertise across services and sectors, where feasible.

## 3. Actively involve the young person

- Include the young person in all stages of transition planning and decision-making.

## 4. Include parents/caregivers

- Involve parents/caregivers with the young person’s consent.
- Respect the young person’s choice of support person.
- Provide guidance and support to families during the shift in responsibilities.

## 5. Assign a transition coordinator

- Designate a consistent point of contact within the care team.
- Involve navigation services to streamline pathways and reduce barriers.

## 6. Provide respectful, person-centred care

- Ensure respectful engagement with young people and their families.
- Support informed choices regarding destination services.

## 7. Tailor support to developmental needs

- Adapt planning to the young person’s maturity, cognitive profile, and social context.
- Coordinate care for those with co-morbid physical health conditions or developmental disorders.

## 8. Adopt a holistic, recovery-oriented approach

- Integrate broader life context into transition planning including education, employment, housing, relationships, and legal circumstances.
- Focus on functional and social outcomes.

## 9. Ensure comprehensive and timely information sharing

- Provide a clear summary of diagnosis, treatment history, and transition details.
- Explain differences between CAMHS and AMHS.
- Ensure materials are accessible in multiple languages.
- Deliver information early for reflection and discussion.
- Provide details of alternative supports if AMHS is not appropriate.

## 10. Utilise appropriate communication tools

- Use digital tools to enhance accessibility and continuity.
- Discuss changes in communication methods in adult services.

# II Transition Decision-Making

## 11. Evaluate ongoing treatment needs

- Assess symptom severity, functional impairment, disorder progression, and comorbidities.
- Promote independence and involve the young person in decision-making.

## 12. Conduct a comprehensive transition need and readiness assessment

- Use validated tools like TRAM.
- Incorporate perspectives from young person, caregiver, and clinician.

## 13. Leverage assessment findings

- Integrate self-reports and proxy assessments into care planning.
- Address discrepancies and provide support.

## 14. Implement regular reassessments

- Monitor changes in symptom severity and treatment needs.
- Adjust care plans accordingly.

## 15. Focus on severity and impairment over diagnosis

- Base decisions on overall impairment and treatment needs.
- Recognise the dimensional nature of disorders.

## 16. Prioritise transition to AMHS for severe mental illness

- Prioritise individuals with severe conditions such as psychosis, bipolar disorder, PTSD, etc, for transition to AMHS.

## 17. Involve families in discharge planning when adult services are unavailable

- Engage families in discharge planning.
- Refer to community-based services if needed.

# III Transition preparation (to AMHS)

## 18. Inform primary care

- With young person’s consent, inform the GP of the transition decision early on.

## 19. Comprehensive preparation

- Develop individualised care and transition plans.
- Provide name of AMHS clinician.
- Prepare for shift from family-centred to person-centred care.
- Include self-management skills training.

## 20. CAMHS relationship closure

- Ensure appropriate (and gradual) closure with CAMHS clinician.
- Review progress and acknowledge achievements.

## 21. Provide accessible records

- Equip young person with documentation about their care in CAMHS.
- Include diagnosis, risks, strengths, care history, medications, and preferences.

## 22. Educate about adult services

- Inform about AMHS expectations.
- Provide coping strategies and resources.

## 23. Promote youth engagement and autonomy

- Encourage active role in care and medication management.
- Support independence and autonomy.

## 24. Peer support

- Facilitate connections with peers who have transitioned.
- Signpost to peer support groups and mentors.

## 25. Support parents’/caregivers’ roles

- Provide guidance and resources for caregivers.
- Connect to carers’ services and support groups.
- Support their attendance at young person’s first AMHS appointment, if appropriate.

## 26. Contingency Plan

- Prepare for delays or unsuccessful transitions.
- Outline interim mental health support strategies.

# IV Continuity of care and service collaboration

## 27. Foster therapeutic relationships

- Involve CAMHS clinicians in AMHS introductions.
- Facilitate joint meetings and shared key workers.

## 28. Flexible pacing

- Tailor transition pace to individual needs.

## 29. Enhance communication and coordination

- Foster collaboration among services.
- Ensure clear handovers and timely assessments.
- Refer promptly to avoid disengagement.

## 30. Ensure effective information sharing

- Transfer key information to receiving service provider.
- Share documentation with young person and parent/caregiver.

## 31. Increase service flexibility

- Advocate for flexible age boundaries.
- Offer extended or parallel care when feasible.

## 32. Provide step-down services

- Offer less intensive support for those not transitioning to AMHS, such as time-limited follow-up appointments to monitor stability.
- Include check-ins and referrals to community-based resources.

# V Care after transition to AMHS

## 33. Enhanced support

- Provide transitional support with AMHS for at least three months.
- Follow up with disengaged young people.

## 34. Clinician consistency

- Ensure same clinician for initial appointments.
- Extend clinician consistency for young people requiring weekly support.

## 35. Choice about parent/caregiver involvement

- Support involvement of parent/carer if desired by the young person.

## 36. Autonomy in treatment decisions

- Respect young person’s treatment decisions.
- Provide support and guidance to navigate these choices confidently.

## 37. Youth-friendly care environments and practices

- Create environments geared toward young adults.
- Offer flexible communication modes.

## 38. Comprehensive evaluation of transition impact

- Use tools like Transition Outcome Measure (TROM) within 4–6 months post-transition to assess the impact of transition.

# VI Managed discharge and follow-up

## 39. Comprehensive assessment and care planning

- Conduct a thorough assessment and develop a care plan.

## 40. Psychoeducation and self-management

- Provide psychoeducation and self-management tools.
- Support independence with suitable apps and symptom trackers.

## 41. Medication management

- Clarify medication oversight.
- Educate about medication and side effects.

## 42. Primary care coordination

- Ensure communication and record transfer to GP.

## 43. Community service/resource linkage

- Support connection to community resources.
- Facilitate contact through joint calls or visits.

## 44. Crisis prevention and management

- Develop crisis plan and re-access pathways.

## 45. Follow-up and monitoring

- Implement structured follow-up through primary care or community services.

## 46. Parent/caregiver support

- Provide ongoing support and education to parents/caregivers.
- Connect them to carers’ services and support groups.

## 47. Pathway for rapid re-entry

- Maintain communication to enable rapid re-entry into services.

# Part 2. Service level guidance

# VII Service improvement and advocacy

## 48. Allocate sufficient budget and resources

- Ensure funding for transition coordinators, clinics, and tools.

## 49. Involve young people in service design

- Engage young people in co-design.
- Create youth-friendly environments.

## 50. Establish shared responsibility and ownership for transition

- Promote shared responsibility for transition outcomes between CAMHS and AMHS.
- Implement collaborative strategies, such as joint clinics and meetings.

## 51. Implement transition protocols

- Develop and update guidelines tailored to local needs and best practices.

## 52. Pre-transition monitoring and planning

- Identify young people approaching age limits.
- Ensure they receive transition readiness assessments.

## 53. Improve service accessibility and navigation

- Locate clinics in accessible areas.
- Maintain updated service information.

## 54. Provide appropriate training and resources

- Train clinicians on legal frameworks, rights, and youth-focused care.
- Deliver joint training across CAMHS and AMHS.

## 55. Support continuous improvement through feedback and follow-up

- Monitor and refine processes using tools like OYOF-TES and OYOF-EOC.

## 56. Collaborate with primary care

- Strengthen collaboration with primary care.
- Explore shared care models and liaison roles.

## 57. Advocate for system improvements

- Identify and document barriers (e.g., rigid timelines and boundaries, resource constraints, long waiting lists).
- Communicate these barriers and their impact through appropriate channels (e.g., team leads, service reviews, policy forums).
- Advocate for solutions such as flexible boundaries, additional resources, and improved funding.
